# Charting the diversity of uncultured viruses of *Archaea* and *Bacteria*

**DOI:** 10.1186/s12915-019-0723-8

**Published:** 2019-12-29

**Authors:** F. H. Coutinho, R. A. Edwards, F. Rodríguez-Valera

**Affiliations:** 1 Evolutionary Genomics Group, Departamento de Produccíon Vegetal y Microbiología, Universidad Miguel Hernández, Campus San Juan, San Juan, 03550 Alicante, Spain; 2 Viral Information Institute, San Diego State University, 5500 Campanile Drive, San Diego, CA 92128 USA

**Keywords:** Viruses, Phylogenomics, Bacteriophages, Metagenomics, Genomics, Classification

## Abstract

**Background:**

Viruses of *Archaea* and *Bacteria* are among the most abundant and diverse biological entities on Earth. Unraveling their biodiversity has been challenging due to methodological limitations. Recent advances in culture-independent techniques, such as metagenomics, shed light on the unknown viral diversity, revealing thousands of new viral nucleotide sequences at an unprecedented scale. However, these novel sequences have not been properly classified and the evolutionary associations between them were not resolved.

**Results:**

Here, we performed phylogenomic analysis of nearly 200,000 viral nucleotide sequences to establish GL-UVAB: Genomic Lineages of Uncultured Viruses of *Archaea* and *Bacteria*. The pan-genome content of the identified lineages shed light on some of their infection strategies, potential to modulate host physiology, and mechanisms to escape host resistance systems. Furthermore, using GL-UVAB as a reference database for annotating metagenomes revealed elusive habitat distribution patterns of viral lineages and environmental drivers of community composition.

**Conclusions:**

These findings provide insights about the genomic diversity and ecology of viruses of prokaryotes. The source code used in these analyses is freely available at https://sourceforge.net/projects/gluvab/.

## Background

Grasping the biodiversity of viruses of *Bacteria* and *Archaea* has been a major challenge within the field of virology. Limitations for viral cultivation and purification associated with the absence of universal marker genes have been major drawbacks in the effort to chart and classify the biodiversity of these viruses [1, 2]. The *Taxonomic* classification system established for viruses of *Bacteria* and *Archaea* was originally based on morphological traits, but genetic studies demonstrated that the major taxa established through this approach are not monophyletic [3–5]. Thus, viral classification and taxonomy have come to rely heavily on comparative genomics. This shift has led the International Committee for the Taxonomy of Viruses (ICTV) to call for a scalable genome-based classification system that can also be applied to uncultured viruses for which no phenotypic data is available [6]. A comprehensive classification system is fundamental for understanding how viruses and their hosts have shaped the evolution of each other and how viruses interact with the ecosystem [7].

Phylogenomic trees and genomic similarity networks incorporate full genomic data for comparison and clustering of viral genomes. Both phylogenomic- and network-based approaches have showed promising results for reconstructing phylogenies and classifying and identifying novel viral taxa [1, 5, 8–10]. These approaches circumvent the biases and limitations associated with morphological data or the use of phylogenetic markers and are easily scalable to thousands of genomes [5, 11]. Network methods rely on the identification of orthologous groups shared among genomes, which can be problematic for viruses due to the rate at which their genes evolve. Additionally, the evolutionary associations among genome clusters identified by network approaches are not explicitly resolved by these methods [5, 12]. Meanwhile, phylogenomic approaches provide trees in which the associations among genomes are easily interpreted under an evolutionary perspective. For these reasons, phylogenomic methods have been the standard approach for reconstructing phylogenies of prokaryotic viruses [1, 8, 11, 13–16]. Previous studies have leveraged this method to investigate the genetic diversity of cultured viruses, but none have done so using all of the uncultured diversity that has recently been described [3, 4, 13, 14, 17–22].

Thousands of novel viral genomic sequences (i.e., complete genomes and genome fragments) were recently discovered through culture-independent approaches, such as shotgun metagenomics, fosmid libraries, single-virus sequencing, and prophage mining [4, 13, 17–20]. These new datasets unraveled an extensive biodiversity that had been overlooked by culture-based approaches. These sequences have the potential to fill many of the gaps in our understanding of the diversity of viruses of prokaryotes. Yet, achieving this goal requires that these genomic sequences are properly organized in a robust evolutionary framework [7]. Here, we applied a phylogenomic approach to chart the diversity of uncultured dsDNA viruses of *Bacteria* and *Archaea* aiming to gain insights on their genetic diversity, evolution, and ecology.

## Results

### Phylogenomic reconstruction

**Fig. 1:**
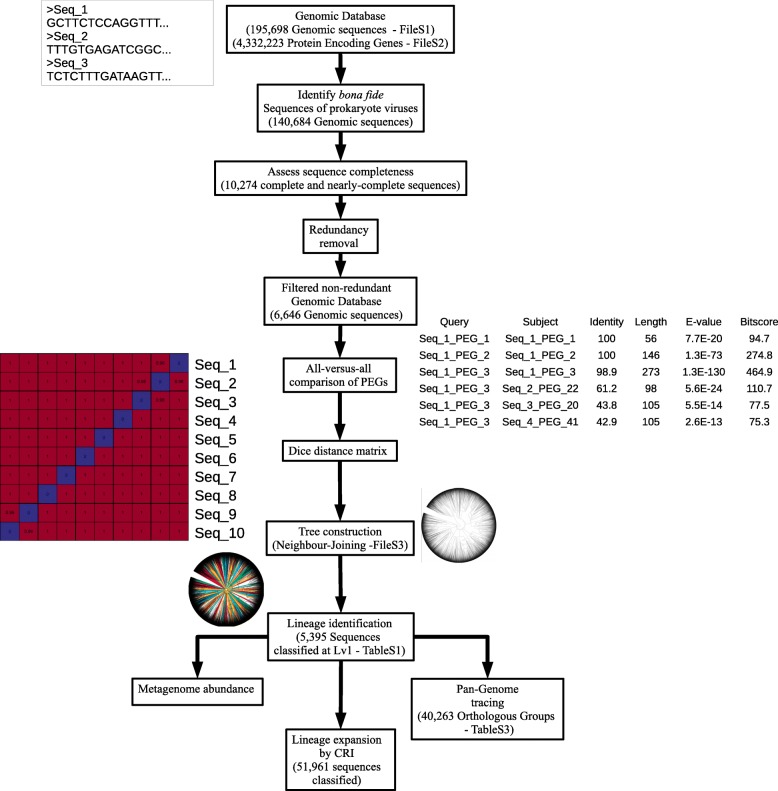
Flowchart summarizing the methodology used to establish GL-UVAB. The initial dataset of genomic sequences consisted of the NCBI RefSeq and viral genomic sequences obtained through culturing independent approaches adding up to 195,698 genomic sequences from which 4,332,223 protein encoding genes (PEGs) were identified. After the initial filtering, 6646 sequences were selected for phylogenomic reconstruction. Dice distances were calculated between this set, and the resulting distance matrix was used for phylogenomic reconstruction through neighbor-joining. The obtained tree was used to identify lineages at three levels, based on minimum node depth: level 1 (node depth equal or above 0.0014, and number of representatives equal or above 20), level 2 (node depth equal or above 0.0056, and number of representatives equal or above 10), and level 3 (node depth equal or above 0.0189, and number of representatives equal or above 3). Lineage abundances were estimated in metagenomic datasets by read mapping. Lineage pan-genomes were determined by identifying clusters of orthologous genes. Finally, sequences that were not included in the original tree were assigned to the lineages by closest relative identification (CRI). Closest relatives were determined based on percentage of matched genes (minimum value of 70%) and average amino acid identity (minimum value of 50%)

An initial database was compiled with all viral sequences from NCBI RefSeq and sequences of uncultured viruses that were discovered across multiple ecosystems using approaches that bypassed culturing. This database amounted to 195,698 viral nucleotide sequences along with associated information of computational host predictions and ecosystem source (Additional file 1). Uncultured viral sequences were filtered to select only those derived from bona fide viruses of *Archaea* and *Bacteria* (see the “Methods” section). Likewise, viral genomes from RefSeq were filtered so that subsequent analysis used only those from dsDNA viruses of *Archaea* and *Bacteria.* Next, redundant sequences were removed as well as those shorter than 10 Kbp that were not annotated as complete or nearly-complete genomes. These filtering steps resulted in a subset of 6646 sequences, out of which 1873 were genomes from NCBI RefSeq. This dataset was used for the phylogenomic reconstruction (Fig. 1, see the “Methods” section for a detailed description of the filtering steps).

**Fig. 2:**
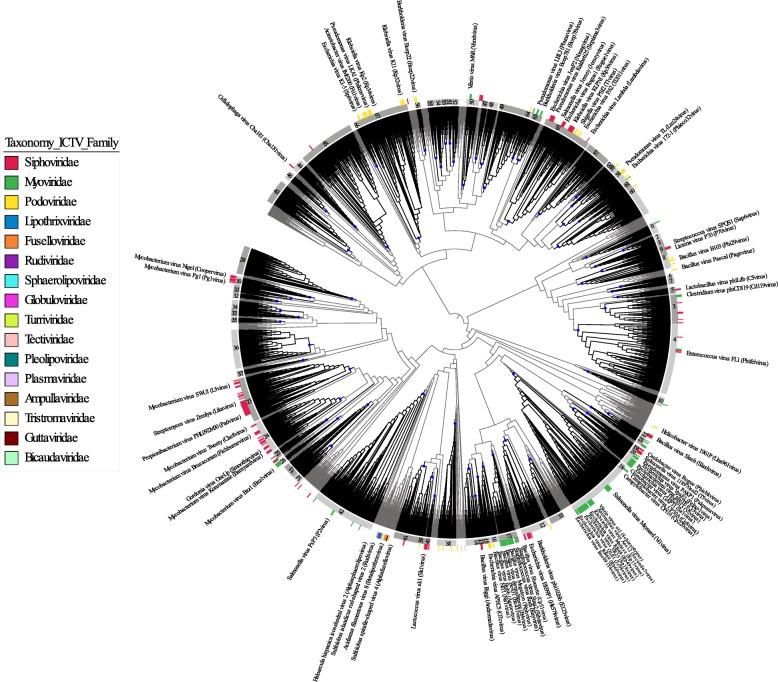
Phylogenomic reconstruction of 6646 viral genomic sequences reveals major lineages of uncultured prokaryotic viruses. The tree was built through neighbor-joining based on Dice distances calculated between viral genomic sequences from both NCBI RefSeq and those reconstructed from metagenomes, fosmid libraries, single virus genomes, and prophages integrated into prokaryote genomes. Tree was midpoint rooted. Branch lengths were omitted to better display tree topology. Each of the 68 level-1 GL-UVAB lineages were highlighted by black colored branches and with their defining nodes indicated by blue dots. Numeric identifiers for the lineages are displayed in the innermost ring within gray strips. The outermost ring depicts the ICTV family-level classification assignments of RefSeq viral genomes that were included in the tree. For reference, a single representative from each ICTV genus was labeled and their *Taxonomic* classification is shown in parentheses

An all-versus-all comparison of the protein sequences encoded in this dataset was performed and used to calculate Dice distances between genomic sequences. Essentially, the Dice distances between a pair of genomic sequences decrease the more proteins that are shared between them and the higher their degree of identity. Finally, the obtained matrix of Dice distances was used to construct a phylogenomic tree through neighbor-joining (Fig. 2 and Additional file 2). The robustness of the tree topology was evaluated through a sub-sampling approach: one hundred phylogenomic trees were reconstructed by randomly removing hits from 5% of the protein encoding genes from the all-versus-all protein search. Next, we measured the frequency in which the nodes from the original tree were present in the re-sampled trees (see the “Methods” section for details). This analysis demonstrated that nodes displayed an average recovery rate of 73.43%. Among all nodes, 96.57% of them were recovered at least once among the re-sampled trees. These figures were obtained when reducing the data used to calculate distances to approximately 90% of the amount used to establish the original tree, demonstrating that tree topology is robust even in the presence of incomplete or fragmented genomes, which might be the case for some of the uncultured viral genomes used. For comparison, we also applied the re-sampling approach to the benchmarking dataset tree of RefSeq viral genomes only. In this dataset, nodes displayed an average recovery rate of 73.22%, and among all nodes, 97.05% of them were recovered at least once among the re-sampled trees. Therefore, the figures of node consistency obtained for the complete tree were similar to those observed for the benchmarking dataset, providing further evidence of the reliability of tree topology.

### Clustering prokaryotic viruses into lineages of closely related genomes

Tree topology confirmed the polyphyletic nature of currently accepted families of prokaryotic viruses, both for the full dataset (Fig. 2) and for the benchmarking (RefSeq only) dataset (Additional file 3). These results corroborate previous findings that showed that the major families within the order Caudovirales (i.e., *Myoviridae*, *Siphoviridae*, and *Podoviridae*) are not monophyletic [1, 3, 4, 10, 11, 23], which justifies the need for a novel classification system based on a phylogenomic approach. We tested different cutoffs of node depth (i.e., distance from the root of the tree) to establish lineages in the benchmarking RefSeq dataset. These cutoffs were scored by the Rand index to determine which values produced maximum agreement with the ICTV classification at the levels of family, sub-family, and genus. Based on these results, a three-step approach was applied to categorize diversity into hierarchical levels of increasing genomic relatedness which respectively correspond to the ranks of family, sub-family, and genus: level 1 (minimum node depth of 0.0014, and number of representatives equal or above 20), level 2 (minimum node depth of 0.0056, and number of representatives equal or above 3), and level 3 (minimum node depth of 0.0189, and number of representatives equal or above 3). The cutoffs for minimum number of representatives were selected so that the higher the level in the hierarchical classification, the higher the number of genomes representing the lineages. We opted for this approach to ensure that level-1 lineages were represented by multiple genomes that displayed a strong signal of genomic relatedness, thus avoiding the establishment of spurious lineages with few genomes, and to account for the presence of incomplete genomic sequences in our dataset. At the first level, 5395 genomic sequences were assigned to 68 lineages (Fig. 2). At the second level, 6198 sequences were assigned to 328 lineages, while at the third level, 5656 sequences were assigned to 407 lineages. This three-level classification system was used to establish the GL-UVAB. The average recovery frequencies derived from the sub-sampling followed by tree reconstruction approach for the nodes used to define level-1, level-2, and level-3 lineages were respectively 31.34%, 73.49%, and 88.26%. These results suggest more reliability of the lineages the lower in the hierarchy. The somewhat lower values observed for level-1 lineages can be attributed to the fact that these lineages were derived from nodes very close to the root of the tree, which are more likely to have their topology affected during the steps of sub-sampling followed by phylogenomic reconstruction.

Genome sequences that were not included in the phylogenomic reconstruction were assigned to the lineage of their closest relative as determined by the average amino acid identity (AAI) and percentage of shared genes. A minimum AAI of 50% and the percentage of matched PEGs of 70% were required for closest relative assignments. Following this step, a total of 51,961 sequences were classified (mean AAI of 75.91% and mean percentage of matched PEGs of 88.31%) to at least one level (Additional file 1), which represents a 22-fold increase in the proportion of classified sequences (both partial and complete genomes) compared to the amount of RefSeq genome sequences of prokaryotic viruses classified by the NCBI taxonomy database at any rank. Importantly, the classification of these genome fragments through this method should be considered tentative, and to be re-evaluated through the phylogenomic approach once the complete genomes are available.

### Correspondence between GL-UVAB lineages and ICTV taxa

We investigated the correspondence between GL-UVAB lineages and the taxa established by the ICTV (Additional file 4). If genomes that belong to the same ICTV taxa are also assigned to the same GL-UVAB lineages, this is an indication of agreement between the two systems. Considering the degrees of similarity selected to establish the GL-UVAB lineages, we compared level-1 lineages to ICTV families, level-2 lineages to ICTV sub-families, and level-3 lineages to ICTV genera respectively. The agreement between the ICTV classification and GL-UVAB system was quantified through the Rand index (The value of this index ranges from 0 to 1. Higher values indicate better agreement between partitions). Level-1 lineages displayed a 0.71 Rand index score when compared to ICTV families, level-2 lineages displayed a 0.95 Rand index score when compared to ICTV sub-families, and level-3 lineages displayed a 0.95 Rand index score when compared to ICTV genera. Overall, these results indicate a strong agreement between the ICTV classification and the GL-UVAB system, specially at the two lowermost levels of the hierarchical classification.

In most cases, the GL-UVAB lineages were composed of genomes derived from only a single ICTV taxon (Additional file 5). Apart from seven cases, all of the 68 level-1 lineages are composed of genomes assigned to a single *Taxonomic* family as defined by the ICTV. The exceptions were most often lineages composed of genomes classified as members from two of the three major families of tailed bacteriophages (e.g., Myoviridae, Podoviridae, and Siphoviridae), which is in agreement with the polyphyletic nature of these taxa [1, 10, 11]. This is also the driving factor behind the lower Rand index observed for level-1 lineages, as our approach identified monophyletic clusters only. Hence, level-1 lineages are equivalent to ICTV families in regard to the degree of similarity among genomes, but with the added advantage of being monophyletic groups. Among level-2 lineages, only a single one encompassed genomes from more than one ICTV defined sub-family (lineage 96 which encompassed members of Ounavirinae and Vequintavirinae). Finally, out of the 88 level-3 lineages that had at least one genome classified by the ICTV at the level of genus, 53 of them are composed of genomes in which members belong to a single ICTV genus. This finding suggests that GL-UVAB level-3 lineages encompass a slightly broader diversity compared to the ICTV genera, which is adequate considering the larger diversity seen among genomes of uncultured viruses. Together, these results demonstrate that the GL-UVAB classification had a strong albeit imperfect agreement with the ICTV established taxonomy and that the cutoffs selected for lineage identification are adequate to derive a classification system based on monophyletic lineages in an automatic manner.

### Targeted hosts and ecosystem sources of GL-UVAB lineages

**Fig. 3:**
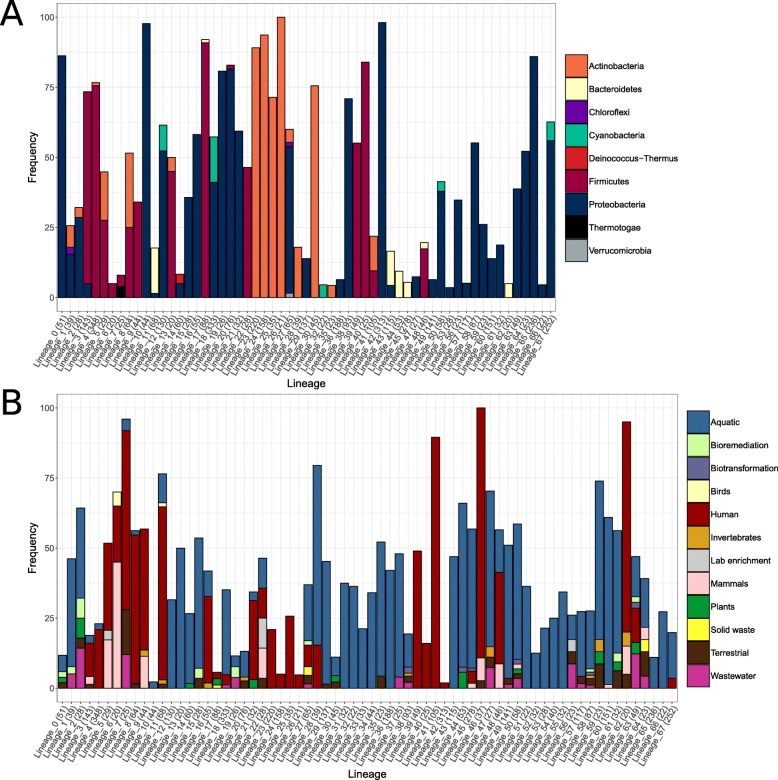
Prevalence of targeted host and ecosystem sources among members of level-1 GL-UVAB lineages assigned through phylogenomic reconstruction. **a** Frequency of infected host phyla across each of the 68 identified lineages. **b** Frequency of ecosystem sources from which viral sequences were obtained across each of the 68 identified lineages. For clarity, only hosts and ecosystems with prevalence equal or above 1% are shown. Numbers in parentheses indicate the total number of genomic sequences assigned to each lineage

GL-UVAB lineages differed regarding host prevalence (Fig. 3a). Out of the 68 level-1 lineages, 33 are predicted to infect a single host phylum, most often *Proteobacteria*, *Firmicutes*, or *Actinobacteria*, while 26 lineages are predicted to infect two or more phyla. Level-3 lineages display the highest levels of host consistency (Additional file 4). Among level-3 lineages with at least one annotated host, 94% of them are predicted to infect a single phylum and 50% are predicted to infect a single genus. Lineages also differed regarding the ecosystem sources from where their members were obtained (Fig. 3b). Nearly all lineages contained members obtained from multiple ecosystems but aquatic and human-associated samples were consistently the main sources of genomic sequences due to the fact that these were the largest datasets in the database of genomic sequences. The trends of host and ecosystem prevalence observed for the expanded lineages established by closest relative identification (Additional file 6) were consistent with those obtained from the lineages defined solely through the phylogenomic tree, further corroborating the validity of these findings.

We sought to further validate these host-lineage associations. Thus, the benchmarking dataset containing a subset of 2069 dsDNA prokaryotic viral genomes from RefSeq was analyzed in parallel for comparison of results. This set of viral genomes with experimentally defined hosts is ideal to observe trends of host prevalence among genomic lineages, without the issues associated with computational host predictions for uncultured viruses. Reconstruction of the phylogenomic tree and lineage identification were performed through the exact same approach used for the complete dataset described above. This analysis of the benchmarking dataset resulted in the identification of 18 level-1 lineages, 48 level-2 lineages, and 132 level-3 lineages (Additional file 3). Among the level-3 lineages of the benchmarking dataset, 126 (95%) are composed of genomes that infect within the same host phylum, corroborating our finding that GL-UVAB lineages constitute cohesive groups regarding their targeted hosts.

**Fig. 4:**
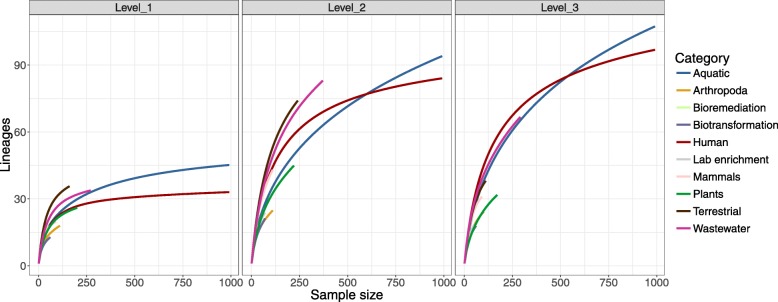
Rarefaction curves depicting the diversity of GL-UVAB lineages across ecosystems. The *X* axis displays the number of randomly sampled genomic sequences from each ecosystem. The *Y* axis depicts the total number of lineages to which these sequences belong to. Each panel represents a different level of the GL-UVAB classification system

Next, we analyzed the contribution of each ecosystem as a source of GL-UVAB lineages. Rarefaction curves revealed that our dataset saturated the diversity of level-1 lineages only in aquatic and human-associated ecosystems (Fig. 4). Nevertheless, the curves for level-2 and level-3 lineages and for the level-1 lineages among other ecosystems did not plateau, suggesting that more of these lineages are still to be discovered and categorized across various ecosystems. The shapes of these curves suggested that aquatic, terrestrial, wastewater, and human-associated ecosystems are among those with the largest diversity of lineages at every level, meaning that these habitats have a high potential for discoveries of novel lineages.

### GL-UVAB lineages display unique patterns of habitat distribution and pan-genome content

**Fig. 5:**
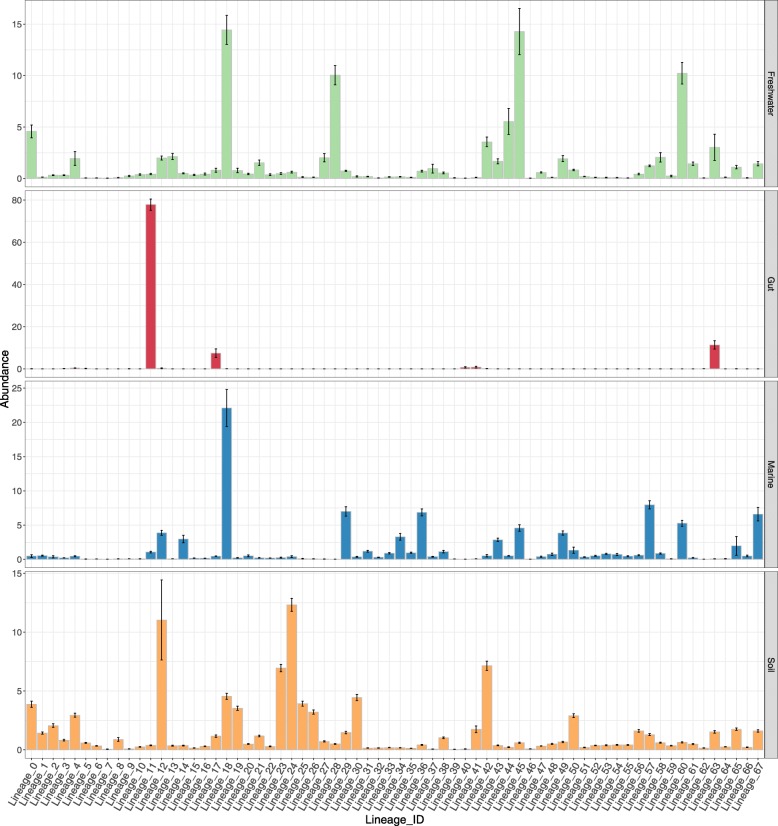
Abundance patterns of GL-UVAB level-1 lineages across habitats. The *Y* axis displays the average and standard errors of the relative abundances (i.e., percentages of the mapped reads) of GL-UVAB level-1 lineages across metagenomes and metaviromes from marine, freshwater, human gut, and soil ecosystems

The observed differences in host preference and ecosystem source among lineages led us to investigate the applicability of GL-UVAB as a reference database for deriving abundance profiles from metagenomes. We analyzed the abundances of 68 GL-UVAB level-1 lineages across metagenomes from marine, freshwater, soil, and human gut samples (Fig. 5). Lineages 18, 57, and 29 were the most abundant in marine samples, in agreement with the high prevalence of *Cyanobacteria* and *Proteobacteria* as their hosts (Fig. 3a). Meanwhile, the lineages 18, 45 (which mostly infects *Bacteroidetes*), and 60 (mostly infects *Alphaproteobacteria*) were the most abundant among freshwater samples. In temperate soil samples, the most abundant lineages were 24 (*Actinobacteria*), 12 (*Gammaproteobacteria*), and 42 (*Gammaproteobacteria*). Finally, human gut samples were dominated by lineages 11 (*Bacteroidetes*), 63 (*Gammaproteobacteria*), and 17 (*Firmicutes*).

**Fig. 6:**
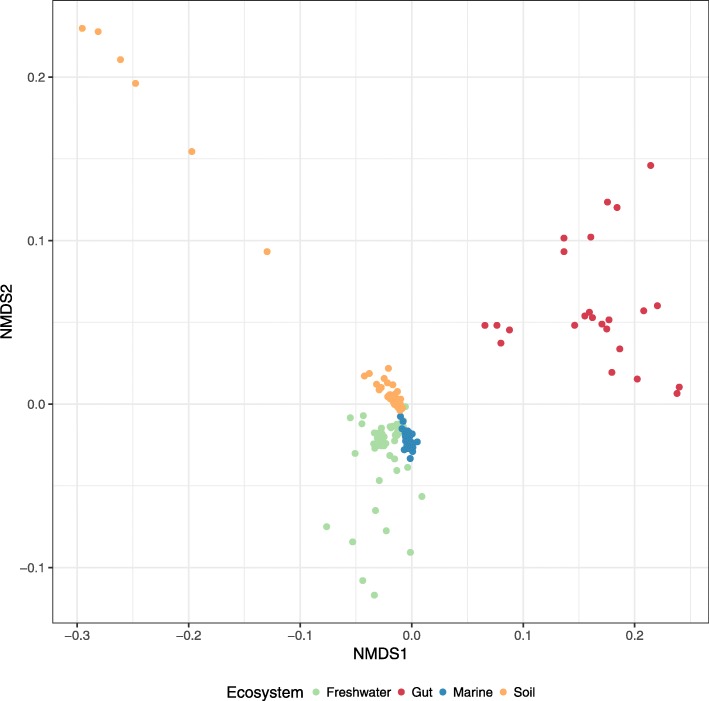
Non-metric multidimensional scaling analysis of metagenomes and metaviromes from marine, freshwater, human gut, and soil ecosystems. Euclidean distances between samples were calculated based on the relative abundances of GL-UVAB level-1 lineages

Exploratory analyses are often performed in studies of viral metagenomics to compare samples based on community composition [24, 25]. Yet these analyses are severely hampered because the majority of the reads from viral metagenomes cannot be assigned a *Taxonomic* classification using databases that encompass only cultured viruses [17]. As a proof-of-principle, the abundances of level-1 lineages across metagenomes were used to compare samples through non-metric multidimensional scaling (Fig. 6). This analysis revealed a clear distinction of samples according to ecosystems. Environmental samples (marine, freshwater, and soil) were roughly separated from gut samples by NMDS1. Meanwhile, aquatic and soil samples were separated by NMDS2. Finally, marine and freshwater samples were separated by NMDS1 as well. These patterns are in agreement with recent findings that demonstrated that, in the global scale, saline/non-saline and free-living/host-associated prokaryote microbiomes have major differences in community composition [26]. Our data reveals that viral communities follow similar trends. Interestingly, this clear distinction between ecosystems could not be observed when annotating these same samples using the ICTV family-level classification as a reference, likely because GL-UVAB covers a much broader diversity of genomic sequences.

Next, we inspected the pan-genome of the identified lineages by clustering their protein encoding genes into orthologous groups (OGs). A total of 40,263 OGs containing at least three proteins were identified. These OGs displayed a sparse distribution, i.e., were only detected in a small fraction of genomic sequences within lineages (Additional file 7), which is likely associated with the fact that not all of the genomes included in this analysis were complete. The most conserved OGs encoded functions associated with nucleic acid metabolism and viral particle assembly. Few OGs encoded putative auxiliary metabolic genes (AMGs), and those where never shared by all the members of a lineage. A total of 1376 promiscuous OGs, present in the pan-genome of three or more level-1 lineages, were identified.

## Discussion

Despite their accelerated pace of evolution and extensive horizontal gene transfer, viruses of prokaryotes can be delineated into genetically cohesive lineages [27]. But only a small fraction of prokaryotic viruses can be cultivated through currently available laboratory techniques. This limitation has left many gaps in our understanding of their biodiversity. The results presented here help to bridge these gaps by leveraging on a large dataset of viral genomic sequences obtained without cultivation from multiple ecosystems. Our findings differ from previous attempts to chart diversity of viruses of *Bacteria* and *Archaea* in several aspects. First, our approach included thousands new genomic sequences of uncultured viruses that have recently been described, while previous phylogenomic analyses have often been restricted to genomes of cultured isolates only [1, 8, 10, 11, 15]. Second, our method was based on a phylogenomic tree which inferred evolutionary associations among viral genomic sequences. Thus, it differs from network-based methods that have recently been proposed for the classification of viruses [5, 9, 12, 28]. Phylogenomic trees explicitly resolve the evolutionary associations among viral genomes which is a major advantage over network-based approaches. Third, not only we provide a phylogeny but also a genome-based classification system encompassing a large diversity of viruses of *Bacteria* and *Archaea*, of an extension unlike any seem before. Our genome-based classification system was shown to be consistent with the *Taxonomic* classification established by the ICTV. Therefore, our approach re-capitulates the currently accepted taxonomy of prokaryotic viruses, with the added advantage to scale to thousands of sequences. Analysis of 6646 genomic sequences including gene calling, Diamond search, Dice distance calculation, phylogenomic reconstruction, and lineage identification took 159 min on a Dell PowerEdge R815 server using 64 processors, demonstrating that our approach can be scaled to even larger datasets encompassing thousands of genomic sequences. Therefore, GL-UVAB was shown to be a valuable tool to investigate the phylogeny of viruses of prokaryotes and to assess and expand the *Taxonomic* classification of uncultured viruses for which only genomic data is available.

The cutoffs used for defining lineages were chosen to classify as many sequences as possible while maintaining cohesiveness within lineages regarding similarity between genomes, targeted hosts, and *Taxonomic* classification as defined by the ICTV. These goals were achieved, as the GL-UVAB lineages are formed by groups of closely related genomic sequences which was reflected in their targeted hosts (Fig. 3a), pan-genome content (Additional file 7), and *Taxonomic* classification (Additional file 4). GL-UVAB was conceived to be an evolving system. We encourage researchers to adapt the GL-UVAB approach to suit the needs of the specific questions under investigation. For example, performing species-level clustering would require node depth cutoffs even higher than those used to delineate level-3 lineages. Importantly, the lineage identification step is dependent on the cutoffs selected for minimum node depth and number of representatives. When reproducing or expanding these analyses, the use of different cutoffs is likely to affect the results. Specifically, lowering either of these values will lead to an increase in the numbers of lineages identified, while raising them will have the opposite effect.

The lack of genes universally shared by viral genomes leads to a difficulty in estimating genomic distances between highly divergent genomes. This trait leads to lower values of the estimated robustness (i.e., recovery rates) of the identified viral lineages, specially those closer to the root of the tree (e.g., level-1 lineages). This is not a limitation associated with our strategy in particular but rather an issue shared by all approaches of viral phylogenomics [10, 11]. Because of that, we chose not to remove lineages based on their degree of estimated robustness. Nevertheless, it is important to keep in mind that those lineages that displayed lower recovery rates are more likely to not be supported by alternative approaches of viral phylogenomics (Additional file 8). Thus, caution is advised when considering the validity of these lineages, as well as any subsequent analysis derived from them. Potential errors when clustering genomes into viral lineages are expected to be propagated into downstream analysis of biogeographical patterns, pan-genome content, and host and ecosystems prevalence, and therefore, these results should be interpreted with care, specially for the level-1 lineages which displayed the lowest degree of robustness.

The consistency of the targeted hosts among lineages identified with our phylogenomic approach suggests that the assignment to GL-UVAB lineages provides a rough estimate of the hosts of uncultured viruses. This is of fundamental importance, considering the growing diversity of viral genomes discovered from metagenomic datasets for which no host information is initially available [29, 30]. Host prevalence analysis indicated that approximately half of the level-1 lineages are capable of infecting more than a single host phylum (Fig. 3a). The ability to interact with the molecular machinery of the host is a major driver of the evolution of prokaryotic viruses. Thus, closely related genomic sequences (that belong to the same lineages) likely have undergone similar evolutionary pressures that ensure host infectivity, leading to the observed pattern of higher host consistency among the lowest level of hierarchical classification (i.e., level-3 lineages). Meanwhile, the ability of some lineages to infect across multiple host phyla is likely an indication of the high level of genomic plasticity of viruses that allows them to evolve to infect new organisms that are not closely related to their original hosts.

The abundance patterns observed for the GL-UVAB lineages (Fig. 5) are a reflection of their distinctive trends of host prevalence (Fig. 3a). As expected, the GL-UVAB lineages that dominated at each ecosystem often targeted taxa that are the most abundant at these habitats [31, 32], e.g., lineages that target *Proteobacteria* and *Cyanobacteria* at aquatic samples and lineages that target *Bacteroidetes* and *Firmicutes* in the human gut. Although this observation might seem obvious, it does not emerge when using cultured viral genomes for the *Taxonomic* annotation of metagenomes. Instead, the same taxa are often observed with similar abundance patterns regardless of the ecosystem sampled. This occurs because established taxa have no discernible host or ecosystem preferences and because much of viral diversity is not encompassed by viral taxonomy [16, 33, 34]. Thus, the cohesiveness of GL-UVAB lineages regarding phylogeny, host preference, and ecology allows for meaningful habitat-taxa associations to be observed. In addition, we demonstrated that the GL-UVAB system can be used for the annotation of viral metagenomes to reveal important trends of viral community composition, highlighting the usefulness of this system for studies of viral ecology.

A detailed investigation of the pan-genome content of the level-1 lineage 18 revealed some of the strategies applied by these viruses during infection. This lineage was among the dominant group in both freshwater and marine samples and infects *Cyanobacteria* and *Proteobacteria.* The pan-genome of lineage 18 includes OGs encoding high-light inducible proteins, photosystem II D1 proteins, and a transaldolase. These proteins are involved in photosynthesis and carbon fixation pathways [35]. Therefore, the success of this group across aquatic ecosystems might be linked to their capacity to use such proteins as AMGs to modulate the metabolism of their Cyanobacterial hosts during infection, redirecting it to the synthesis of building blocks to be used for the assembly of novel viral particles [35].

The promiscuous distribution observed for multiple OGs could be the result of the positive selection of these genes following events of horizontal gene transfer (HGT). Indeed, promiscuous OGs often encoded proteins that might confer advantages during infection. Eight of them encoded thymidylate synthase, a protein involved in nucleotide synthesis. Meanwhile, two promiscuous OGs encoded the *PhoH* protein, which mediates phosphorus acquisition in nutrient-deprived conditions. These findings suggest a selective pressure favoring the acquisition of genes that allow viruses to modulate host metabolism towards the production of nucleic acids to be used for the synthesis of progeny DNA [35]. Multiple methylases were identified among promiscuous OGs. Viruses use these proteins to protect their DNA from host restriction modification systems [36]. Prokaryotes can acquire restriction modification systems through HGT [37], and our data suggests that viruses also benefit from HGT by acquiring novel methylases that allow them to escape these systems. Finally, lysins (e.g., peptidases and amidases) were a common function among promiscuous OGs. This finding is surprising because lysins are believed to be fine-tuned for the specific structure of host cell wall [38, 39]. Acquisition of novel lysins might help viruses to expand their host spectra or as a mechanism to ensure infectivity following the emergence of resistance mutations that lead to alterations in the structure of the host cell wall.

In conclusion, by analyzing thousands of uncultured viral nucleotide sequences, we were able to categorize the diversity of these biological entities. This was achieved by identifying lineages of uncultured viruses through a robust and scalable phylogenomic approach. Analyzing host and source prevalence, pan-genome content, and abundance in metagenomes painted a more accurate picture of viral biodiversity across ecosystems, highlighted the potential for discoveries across different habitats, and shed light on ecological drivers of viral community composition. We made available the source code [40] along with all the data necessary to replicate our analyses (Additional file 9: File S1). Thus, the community can easily expand GL-UVAB and apply this approach to their specific demands. Future studies will continue to shed light on viral diversity across our planet’s many ecosystems. Our work provides the initial steps for a genome-based classification of these yet undiscovered evolutionary lineages, providing a solid framework to investigate the biology of prokaryotic viruses in the future.

## Methods

### Viral genome database

The NCBI RefSeq dataset was used as a starting set of reference viral genomic sequences. Host information for these sequences was retrieved from GenBank files, and their *Taxonomic* classification was obtained both from the NCBI Taxonomy database and from the ICTV [41]. Additionally, genomic sequences (i.e., complete and partial genomes) were compiled from studies that used high-throughput approaches to obtain viral genomes through culture-independent analysis. These sequences of uncultured viruses were obtained from environmental metagenomes and metaviromes [3, 14, 17, 18, 20–22], fosmid libraries of Mediterranean viruses [4, 13], single virus genomes [42], and prophages integrated into prokaryotic genomes [19]. We also retrieved the associated metadata, which included information on putative hosts and ecosystem source.

This dataset contained both RefSeq and uncultured viral sequences (henceforth referred to as Vir_DB_Nuc) and contained a total of 195,698 viral nucleotide sequences (Additional files 1 and 9). Protein encoding genes (PEGs) were predicted from Vir_DB_Nuc using the metagenomic mode of Prodigal [43], which identified 4,332,223 protein sequences (henceforth referred to as Vir_DB_Prot, Additional file 10). The Vir_DB_Prot dataset was queried against the NCBI-nr protein database using Diamond [44] for *Taxonomic* and functional annotation.

### Sequence pre-filtering

Identifying viral sequences within metagenomic and metaviromic datasets can be problematic. Because each study used different strategies to achieve that goal, we pre-filtered sequences from Vir_DB_Nuc to ensure that only bona fide viral sequences were included in downstream analyses. We considered as bona fide viral sequences those complete and partial genomic sequences that displayed a strong viral signal. This viral signal was assessed in three distinct steps that relied on sequence homology. First, the Vir_DB_Prot dataset was queried against the prokaryotic virus orthologous groups (pVOGs) [45] protein database using Diamond [44] (more sensitive mode, BLOSUM45 matrix, identity ≥ 30%, bitscore ≥ 50, alignment length ≥ 30 amino acids, and *e* value ≤ 0.01). Each orthologous group in the pVOGs database is assigned a viral quotient which ranges from 0 to 1. The more specific to viral genomes the orthologous group is the closer to one this viral quotient is, meaning that groups with a quotient of 1 are found exclusively in viral genomes and were never detected in genomes of *Bacteria* or *Archaea*. For each genomic sequence in our Vir_DB_Nuc, we calculated the percentage of proteins mapped to the pVOGs database and their added viral quotient (AVQ). The AVQ was defined as the sum of the individual viral quotients of the best hits of each protein from the viral genomic sequences mapped to the pVOGs database. Also, we queried proteins from Eukaryotic virus genomes in Vir_DB_Nuc against the pVOGs database using DIAMOND as described above. Any pVOGs that matched proteins from Eukaryotic viruses were excluded from the pVOGs database for subsequent searches, meaning that they were not considered when calculating AVQ scores to identify bona fide viral sequences. Sequences with 20% or more of the proteins mapped to the pVOGs database and with an AVQ equal to or greater than 5 were classified as bona fide genomic sequences of prokaryotic viruses. These cutoffs were determined by analyzing both eukaryotic and prokaryotic Refseq viral genomes, and selected to maximize precision and recall of the recruitment step. This initial round of recruitment yielded 26110 genomic sequences (Vir_DB_Nuc_R1). Second, proteins from the Vir_DB_Nuc_R1 dataset were used as bait for a second recruitment round. The remaining protein sequences (which were not recruited in the first round) were queried against Vir_DB_Nuc_R1 through Diamond as described above. Genomic sequences from which at least 20% of the derived proteins mapped to a single genome from Vir_DB_Nuc_R1, yielding a minimum of three protein matches, were recruited to Vir_DB_Nuc_R2 (78,295 genomic sequences). Third, a step of manual curation was performed, which recruited mostly long sequences with high AVQ that did not match the percentage criteria of the automatic recruiting steps due to their high number of encoded proteins. This step recruited a total of 6420 genomic sequences (Vir_DB_Nuc_R3).

We benchmarked the accuracy of the automatic recruiting steps with two datasets. First, a subset of Vir_DB_Nuc comprised only of the viral genomes from RefSeq was run through the recruitment pipeline using the same criteria described above. None of the 7036 eukaryotic viruses were recruited by the pipeline (i.e., 100% precision) and 2136 out of 2297 prokaryotic viruses were correctly recruited (i.e., 92.99% recall). We also benchmarked the filtering pipeline with a dataset of 897 Gbp of genome sequence data derived from the NCBI RefSeq prokaryote genomes spanning 880 genera from 35 phyla. Sequences were split into fragments of 5, 10, 15, 20, 25, 50, and 100 Kbp to mimic metagenomic scaffolds. Using the filtering criteria described above and a subsequent length filtering for sequences longer than 30 Kbp would recruit only 109 sequences (0.36%), all of which displayed homology to the prophage sequences described by Roux et al. [19].

In addition*,* we confirmed the viral origin of the recruited sequences by analyzing them through VirSorter [19] and VirFinder [46]. Overall there was a strong agreement between the pVOGs approach and the two aforementioned methods: 90.3% of all the sequences recruited based on pVOGs scores were also annotated as bona fide viruses by VirSorter (categories 1, 2, 4, and 5) and/or VirFinder (score ≥ 0.6 and *p* value ≤ 0.05). We observed that a total of 27,562 sequences were identified as viral by VirSorter but not by our automatic recruitment approaches, suggesting this tool has a better recall for identifying viral sequences. Thus, we included those sequences as bona fide viral sequences in our dataset as well (Vir_DB_Nuc_R4). The remaining sequences (that were not recruited to Vir_DB_Nuc_R1, Vir_DB_Nuc_R2, Vir_DB_Nuc_R3 or Vir_DB_Nuc_R4) could be viral, but since they did not display a clearly viral signature they were excluded from the subsequent steps of phylogenomic reconstruction.

### Sequence completeness

The publications that originally described the aforementioned sequences also determined genome completeness, often by searching for overlapping sequence ends or by identifying synteny and homology with complete viral genomes. Completeness information was retrieved from the metadata in the original publications. RefSeq genomes were tagged as complete if their description field included the term “complete genome.” We also performed an additional search for circular sequences by identifying overlapping ends through VirSorter [19]. Next, we queried the proteins from all sequences in Vir_DB_Nuc annotated as complete against those derived from genomes that were not tagged as complete. This search was performed through Diamond (set to more sensitive mode, identity ≥ 30%, bitscore ≥ 30, alignment length ≥ 30 amino acids, and *e* value ≤ 0.01). If 70% or more of all the proteins of a single complete genome “A” could be mapped to a given sequence “B,” then that genome “B” was tagged as nearly-complete (provided that genome “B” had a length of at least 10 Kbp).

### Phylogenomic reconstruction

Phylogenomic reconstruction was performed using a subset of genomes from Vir_DB_Nuc that included all dsDNA RefSeq viral genomes annotated as complete or nearly-complete for which the host Domain was either *Bacteria* or *Archaea* and the uncultured bona fide prokaryotic viruses from Vir_DB_Nuc_R1, Vir_DB_Nuc_R2, Vir_DB_Nuc_R3, and Vir_DB_Nuc_R4 with a length equal or greater than 10 Kbp and annotated as a complete or nearly-complete viral genomes. These criteria were established to minimize any issues that might arise from the use of incomplete genomes in the phylogenomic reconstruction. Genome sequences were clustered with CD-HIT [47] using a cutoff of 95% nucleotide identity and minimum 50% coverage of the shorter sequence to remove redundant sequences. The non-redundant dataset contained 6646 viral nucleotide sequences that were used for phylogenomic reconstruction (Vir_DB_Phy). Distances between genomic sequences were calculated based on a modified version of the Dice method [4]. First, an all-versus-all comparison of the PEGs derived from the Vir_DB_Phy dataset was performed through Diamond [44] (more sensitive mode, identity ≥ 30%, bitscore ≥ 30, alignment length ≥ 30 amino acids, and *e* value ≤ 0.01). Next, distances between genomic sequences were calculated as follows: *D*_AB_ = 1 − (2 × (AB)/(AA+BB)), where AB is the bitscore sum of all the valid protein matches of sequence A against sequence B, while AA and BB are the bitscore sum of all the valid protein matches of sequence A against itself and of all the valid protein matches of sequence B against itself, respectively. The more homologous proteins are shared between A and B, and the higher the percentage of identity between these homologous proteins, the closer to zero the value of *D*_AB_ will be. Nonhomologous proteins should produce no matches when comparing sequence A against B, but will match with themselves when comparing A against A and B against B. Therefore, when estimating *D*_*AB*_, nonhomologous proteins are penalized, increasing the value of *D*_AB_*.* The obtained Dice distances matrix was used as input to build a phylogenomic tree through neighbor-joining algorithm [48] implemented in the Phangorn package of R. The obtained tree was midpoint rooted (Additional file 11). In parallel, a benchmarking dataset comprised of 2069 genome sequences of dsDNA viruses of *Archaea* and *Bacteria* from the NCBI RefSeq database was also subjected to phylogenomic reconstruction. The steps for distance calculation, tree construction, and lineage identification were performed exactly as described above for the full dataset.

### Tree topology validation by re-sampling

A re-sampling approach was applied to test the consistency of tree topology. First, 5% of the proteins encoded in the genomic sequences used to build the tree were randomly selected. Then, distances between genomes were re-calculated after excluding any protein matches from the all-versus-all search in which either the query or subject sequences were selected for exclusion, which removes approximately 10% of all of the original matches. Finally, the obtained distance matrix was used to construct a new tree. This process was repeated over 100 iterations. Next, we measured the frequency in which the nodes from the original tree were present in the re-sampled trees. This strategy was applied for both the Vir_DB_Phy and the RefSeq benchmarking dataset. For reference, we also performed this analysis using different values of percentage of removed proteins (1–20%) and 50 iterations.

### Lineage identification

First, we sought to establish cutoffs for lineage identification that produced maximum agreement with the ICTV *Taxonomic* classification. Thus, the phylogenomic tree built for the benchmarking dataset was parsed to identify monophyletic clades as candidate lineages based on minimum node depth (i.e., distance from the root of the tree). During this testing step, the values of minimum node depth cutoff ranged from 0.0001 to 0.2, incremented in steps of 0.0001. Next, the lineages identified for each cutoff value were compared to the ICTV classification at the ranks of family, sub/family, and genus, and scored according to the Rand index. The cutoffs that yielded the highest Rand index scores were selected as the ideal ones for identifying lineages de novo for each rank in the full dataset (Vir_DB_Phy). Thus, lineage identification was performed by parsing the Vir_DB_Phy phylogenomic tree to identify monophyletic clades that matched the established criteria based on minimum node depth, and for a minimum number of representatives. Lineages were identified de novo in three steps, aimed at capturing diversity into levels of increasing genomic relatedness: level-1 (node depth equal or above 0.0014, and number of representatives equal or above 20), level-2 (node depth equal or above 0.0056, and number of representatives equal or above 3), and level-3 (node depth equal or above 0.0189, and number of representatives equal or above 3). To trace the pan-genomes of the identified lineages, the proteins derived from 6646 genomic sequences in Vir_DB_Phy were clustered into orthologous groups using the orthoMCL algorithm [49] implemented in the Get_Homologues pipeline [50]. The MCL inflation factor was set to 1, and all other parameters were set to default.

### Lineage expansion by closest relative identification

Sequences that did not pass the initial length and redundancy filters to be included in the phylogenomic tree were assigned to the lineages of their closest relatives. Closest relatives were defined as the sequence with the highest percentage of matched protein encoding genes (PEGs) as detected by Diamond searches. A minimum AAI of 50% and the percentage of matched PEGs of 70% were required for closest relative assignments. Potential ties were resolved by choosing the closest relative with the highest average amino acid identity (AAI) value.

### Lineage abundance in metaviromes and metagenomes

The abundances of Vir_DB_Nuc sequences were estimated in viral metagenomes (viromes) from the following ecosystems: marine epipelagic samples [51], healthy human gut [52], and freshwater lakes [53], and because no large-scale viromes of mesophilic soils were available, we used cellular metagenomes from this ecosystem [54, 55]. Sequencing reads from these metagenomes and metaviromes were retrieved from the European Nucleotide Archive or NCBI Short Read Archive. Subsets of 20 million R1 reads from each sample were mapped to Vir_DB_Nuc using Bowtie2 [56] using the sensitive-local alignment mode. Lineage abundances across samples were calculated by summing the relative abundances of individual genomic sequences according to their assigned lineages.

## Supplementary information

**Additional file 1: Table S1.** Table containing detailed information of all the genomic sequences from Vir_DB_Nuc analyzed in this study, including sequence identifier, NCBI access number, original dataset, sequence length, number of identified PEGs, *Taxonomic* classification (NCBI and ICTV), ecosystem source and host *Taxonomic* classification and affiliation to the identified GL-UVAB lineages at three hierarchical levels. The pVOGs_Perc_Matched field corresponds to the percentage of proteins derived from a sequence that matched the pVOGs database and the pVOGs_VQ_Score corresponds to the added viral quotient (AVQ) of these matches. The Bona_Fide_Phage_Homology_CR_ID corresponds to the identifier of the closest relative identified for a given sequence among those of Vir_DB_Nuc_R1. Bona_Fide_Phage_Homology_CR_Hits represents the absolute number of protein matches to the closest relative and Bona_Fide_Phage_Homology_CR_Perc_Matched represents the percentage of proteins in a sequence covered by these matches.

**Additional file 2: Figure S1.** Phylogenomic reconstruction of 6646 viral genomic sequences. The tree was built through Neighbor-Joining based on Dice distances calculated between viral genomic sequences from both NCBI RefSeq and those reconstructed from metagenomes, fosmid libraries and prophages integrated into prokaryote genomes. The tree was midpoint rooted. To better display higher-order associations between lineages, nodes were collapsed according to their Level-1 lineage assignments or if all the leaves in a node were not assigned to any lineages.

**Additional file 3: Figure S2.** Phylogenomic reconstruction of 2069 genomes of dsDNA viruses of Archaea and Bacteria from RefSeq. The tree was built through Neighbor-Joining based on Dice distances calculated between complete prokaryotic viral genomes from NCBI RefSeq. The tree was midpoint rooted. The innermost ring displays ICTV family level *Taxonomic* classification, the middle ring displays ICTV subfamily level *Taxonomic* classification, and the outermost ring displays classifications into lineages identified for the benchmarking dataset.

**Additional file 4: Table S2.** Prevalence of targeted hosts, ecosystem and dataset sources, *Taxonomic* classification and completeness of genomic sequences among the three levels of GL-UVAB lineages.

**Additional file 5: Figure S3.** Concordance between the ICTV taxonomy and the GL-UVAB classification system. A**)** Bar plots depicting the prevalence of ICTV family level genome classifications among the Level-1 GL-UVAB lineages. B**)** Bar plots depicting the prevalence of ICTV sub-family level genome classifications among the Level-2 GL-UVAB lineages. C) Heatmap depicting the prevalence of ICTV genera (columns) level classification among Level-3 GL-UVAB lineages (rows). Within squares are depicted the absolute number genomes from a genus assigned to a given lineage, while the color gradient represents the percentage of genomes from a genus assigned to a given lineage. To facilitate visualization rows and columns were clustered based on euclidean distances.

**Additional file 6: Figure S4.** Prevalence of targeted host and ecosystem sources among Level-1 GL-UVAB lineages assigned through phylogenomic reconstruction and lineage expansion by closest relative identification. A) Frequency of infected host phyla across each of the 68 identified lineages. B) Frequency of ecosystem sources from which viral sequences were obtained across each of the 68 identified lineages. For clarity, only hosts and ecosystems with prevalence within a lineage equal or above 1% are shown. Numbers in parentheses indicate the total number of genomes assigned to each lineage after the step of classification through closest relative identification.

**Additional file 7: Table S3.** Table describing the prevalence of OGs across GL-UVAB Level-1 lineages with associated *Taxonomic* and functional annotation. Only OGs detected in at least 3 members of a lineage are shown.

**Additional file 8: Table S4.** Characteristics of the nodes of the tree depicted in Fig. 1, including depth, height, and robustness measured with varying values of percentage of proteins removed during sub-sampling with 50 replicates for each value.

**Additional file 9: File S1.** Link to download the multifasta file containing the 195,698 viral genomic sequences from Vir_DB_Nuc analyzed in this study.

**Additional file 10: File S2.** Link to download the multifasta file containing the 4,332,223 protein encoding gene sequences predicted from Vir_DB_Nuc.

**Additional file 11: File S3.** Phylogenomic tree used to define GL-UVAB lineages. The tree was constructed with the Neighbor-Joining algorithm based on Dice distances calculated between 6,646 genomic sequences.

## Data Availability

The datasets supporting the conclusions of this article are included within the article and its additional files. The sequence data used in this article can be downloaded from a public repository through the links provided in Additional files 10 and 11. The source code for GL-UVAB is freely available for unrestricted usage under the GPL-3.0 license at SourceForge at https://sourceforge.net/projects/gluvab/ [40]. The operating system is platform independent, and the code was written in Perl, requiring Perl (v5.14.2 or higher), BioPerl, Prodigal (v2.60 or higher), DIAMOND, (v0.9.14 or higher), and R (v3.2.5 or higher). Analyses were carried out using version 0.6.

## References

[1] Rohwer F, Edwards R (2002). The phage proteomic tree: a genome-based taxonomy for phage. J Bacteriol.

[2] Pedulla ML, Ford ME, Houtz JM, Karthikeyan T, Wadsworth C, Lewis JA (2003). Origins of highly mosaic mycobacteriophage genomes. Cell..

[3] Nishimura Y, Watai H, Honda T, Mihara T, Omae K, Roux S (2017). Environmental viral genomes shed new light on virus-host interactions in the ocean. mSphere.

[4] Mizuno CM, Rodriguez-Valera F, Kimes NE, Ghai R (2013). Expanding the marine virosphere using metagenomics. PLoS Genet.

[5] Bolduc B, Bin JH, Doulcier G, You Z-Q, Roux S, Sullivan MB (2017). vConTACT: an iVirus tool to classify double-stranded DNA viruses that infect *Archaea* and *Bacteria*. PeerJ.

[6] Simmonds P, Adams MJ, Benkő M, Breitbart M, Brister JR, Carstens EB (2017). Virus taxonomy in the age of metagenomics. Nat Rev Microbiol.

[7] Kuhn JH, Wolf YI, Krupovic M, Zhang Y-Z, Maes P, Dolja VV (2019). Classify viruses — the gain is worth the pain. Nature..

[8] Nishimura Y, Yoshida T, Kuronishi M, Uehara H, Ogata H, Goto S (2017). ViPTree: the viral proteomic tree server. Bioinformatics..

[9] Bin Jang H, Bolduc B, Zablocki O, Kuhn JH, Roux S, Adriaenssens EM (2019). *Taxonomic* assignment of uncultivated prokaryotic virus genomes is enabled by gene-sharing networks. Nat Biotechnol.

[10] Low SJ, Džunková M, Chaumeil P-A, Parks DH, Hugenholtz P (2019). Evaluation of a concatenated protein phylogeny for classification of tailed double-stranded DNA viruses belonging to the order Caudovirales. Nat Microbiol.

[11] Meier-Kolthoff JP, Göker M (2017). VICTOR: genome-based phylogeny and classification of prokaryotic viruses. Bioinformatics..

[12] Lima-Mendez G, Van Helden J, Toussaint A, Leplae R (2008). Reticulate representation of evolutionary and functional relationships between phage genomes. Mol Biol Evol.

[13] Mizuno CM, Ghai R, Saghaï A, López-García P, Rodriguez-Valera F (2016). Genomes of abundant and widespread viruses from the deep ocean. MBio..

[14] López-Pérez M, Haro-Moreno JM, Gonzalez-Serrano R, Parras-Moltó M, Rodriguez-Valera F (2017). Genome diversity of marine phages recovered from Mediterranean metagenomes: size matters. PLoS Genet.

[15] Adriaenssens EM, Edwards R, Nash JHE, Mahadevan P, Seto D, Ackermann HW (2015). Integration of genomic and proteomic analyses in the classification of the Siphoviridae family. Virology..

[16] Bellas CM, Anesio AM, Barker G (2015). Analysis of virus genomes from glacial environments reveals novel virus groups with unusual host interactions. Front Microbiol.

[17] Coutinho FH, Silveira CB, Gregoracci GB, Thompson CC, Edwards RA, Brussaard CPD (2017). Marine viruses discovered via metagenomics shed light on viral strategies throughout the oceans. Nat Commun.

[18] Roux S, Brum JR, Dutilh BE, Sunagawa S, Duhaime MB, Loy A (2016). Ecogenomics and biogeochemical impacts of uncultivated globally abundant ocean viruses. Nature..

[19] Roux S, Hallam SJ, Woyke T, Sullivan MB (2015). Viral dark matter and virus-host interactions resolved from publicly available microbial genomes. Elife..

[20] Paez-Espino D, Eloe-Fadrosh EA, Pavlopoulos GA, Thomas AD, Huntemann M, Mikhailova N (2016). Uncovering Earth’s virome. Nature..

[21] Vik DR, Roux S, Brum JR, Bolduc B, Emerson JB, Padilla CC (2017). Putative archaeal viruses from the mesopelagic ocean. PeerJ..

[22] Philosof A, Yutin N, Flores-Uribe J, Sharon I, Koonin EV, Béjà O (2017). Novel abundant oceanic viruses of uncultured marine group II Euryarchaeota identified by genome-centric metagenomics. Curr Biol.

[23] Aiewsakun P, Adriaenssens EM, Lavigne R, Kropinski AM, Simmonds P (2018). Evaluation of the genomic diversity of viruses infecting bacteria, archaea and eukaryotes using a common bioinformatic platform: steps towards a unified taxonomy. J Gen Virol.

[24] Tseng C-H, Chiang P-W, Shiah F-K, Chen Y-L, Liou J-R, Hsu T-C (2013). Microbial and viral metagenomes of a subtropical freshwater reservoir subject to climatic disturbances. ISME J.

[25] Williamson SJ, Allen LZ, Lorenzi HA, Fadrosh DW, Brami D, Thiagarajan M (2012). Metagenomic exploration of viruses throughout the Indian Ocean. PLoS One.

[26] Thompson LR, Sanders JG, McDonald D, Amir A, Ladau J, Locey KJ (2017). A communal catalogue reveals Earth’s multiscale microbial diversity. Nature..

[27] Bobay L-M, Ochman H (2018). Biological species in the viral world. Proc Natl Acad Sci.

[28] Shapiro JW, Putonti C (2018). Gene co-occurrence networks reflect bacteriophage ecology and evolution. MBio..

[29] Aylward FO, Boeuf D, Mende DR, Wood-Charlson EM, Vislova A, Eppley JM (2017). Diel cycling and long-term persistence of viruses in the ocean’s euphotic zone. Proc Natl Acad Sci.

[30] Luo E, Aylward FO, Mende DR, DeLong EF (2017). Bacteriophage distributions and temporal variability in the ocean’s interior. MBio..

[31] Sunagawa S, Coelho LP, Chaffron S, Kultima JR, Labadie K, Salazar G (2015). Structure and function of the global ocean microbiome. Science.

[32] Arumugam M, Raes J, Pelletier E, Le Paslier D, Yamada T, Mende DR (2011). Enterotypes of the human gut microbiome. Nature..

[33] Aguirre de Cárcer D, López-Bueno A, Pearce DA, Alcamí A (2015). Biodiversity and distribution of polar freshwater DNA viruses. Sci Adv.

[34] Zablocki O, van Zyl L, Adriaenssens EM, Rubagotti E, Tuffin M, Cary SC (2014). High-level diversity of tailed phages, eukaryote-associated viruses, and virophage-like elements in the metaviromes of Antarctic soils. Appl Environ Microbiol.

[35] Thompson LR, Zeng Q, Kelly L, Huang KH, Singer AU, Stubbe J (2011). Phage auxiliary metabolic genes and the redirection of cyanobacterial host carbon metabolism. Proc Natl Acad Sci.

[36] Samson JE, Magadán AH, Sabri M, Moineau S (2013). Revenge of the phages: defeating bacterial defences. Nat Rev Microbiol..

[37] Oliveira PH, Touchon M, Rocha EPC (2014). The interplay of restriction-modification systems with mobile genetic elements and their prokaryotic hosts. Nucleic Acids Res.

[38] Oliveira H, Melo LDR, Santos SB, Nobrega FL, Ferreira EC, Cerca N (2013). Molecular aspects and comparative genomics of bacteriophage endolysins. J Virol.

[39] Fernández-Ruiz I, Coutinho FH, Rodriguez-Valera F (2018). Thousands of novel endolysins discovered in uncultured phage genomes. Front Microbiol.

[40] Coutinho FH (2019). GLUVAB.

[41] Krupovic M, Dutilh BE, Adriaenssens EM, Wittmann J, Vogensen FK, Sullivan MB (2016). Taxonomy of prokaryotic viruses: update from the ICTV bacterial and archaeal viruses subcommittee. Arch Virol.

[42] Martinez-Hernandez F, Fornas O, Lluesma Gomez M, Bolduc B, de la Cruz Peña MJ, Martínez JM (2017). Single-virus genomics reveals hidden cosmopolitan and abundant viruses. Nat Commun.

[43] Hyatt D, Chen G-L, Locascio PF, Land ML, Larimer FW, Hauser LJ (2010). Prodigal: prokaryotic gene recognition and translation initiation site identification. BMC Bioinformatics.

[44] Buchfink B, Xie C, Huson DH (2015). Fast and sensitive protein alignment using DIAMOND. Nat Methods.

[45] Grazziotin AL, Koonin EV, Kristensen DM (2017). Prokaryotic virus orthologous groups (pVOGs): a resource for comparative genomics and protein family annotation. Nucleic Acids Res.

[46] Ren J, Ahlgren NA, Lu YY, Fuhrman JA, Sun F (2017). VirFinder: a novel k-mer based tool for identifying viral sequences from assembled metagenomic data. Microbiome..

[47] Fu L, Niu B, Zhu Z, Wu S, Li W (2012). CD-HIT: accelerated for clustering the next-generation sequencing data. Bioinformatics..

[48] Saitou NNM (1987). The neighbor-joining method: a new method for reconstructing phylogenetic trees. Mol Biol Evol.

[49] Li L, Stoeckert CJ, Roos DS (2003). OrthoMCL: identification of ortholog groups for eukaryotic genomes. Genome Res.

[50] Contreras-Moreira B, Vinuesa P (2013). GET_HOMOLOGUES, a versatile software package for scalable and robust microbial pangenome analysis. Appl Environ Microbiol.

[51] Brum JR, Ignacio-Espinoza JC, Roux S, Doulcier G, Acinas SG, Alberti A (2015). Patterns and ecological drivers of ocean viral communities. Science..

[52] Minot S, Bryson A, Chehoud C, Wu GD, Lewis JD, Bushman FD (2013). Rapid evolution of the human gut virome. Proc Natl Acad Sci.

[53] Roux S, Chan L-K, Egan R, Malmstrom RR, McMahon KD, Sullivan MB (2017). Ecogenomics of virophages and their giant virus hosts assessed through time series metagenomics. Nat Commun.

[54] Rascovan N, Carbonetto B, Revale S, Reinert MD, Alvarez R, Godeas AM (2013). The PAMPA datasets: a metagenomic survey of microbial communities in Argentinean pampean soils. Microbiome..

[55] Luo C, Rodriguez-R LM, Johnston ER, Wu L, Cheng L, Xue K (2014). Soil microbial community responses to a decade of warming as revealed by comparative metagenomics. Appl Environ Microbiol.

[56] Langmead B, Salzberg SL (2012). Fast gapped-read alignment with Bowtie 2. Nat Methods.

