# Supplementary figures and images for: Charting the diversity of uncultured viruses of Archaea and Bacteria
Source: BMC Biol. 2019 Dec 29;17:109. doi: 10.1186/s12915-019-0723-8 (PMC6936153; doi:10.1186/s12915-019-0723-8)

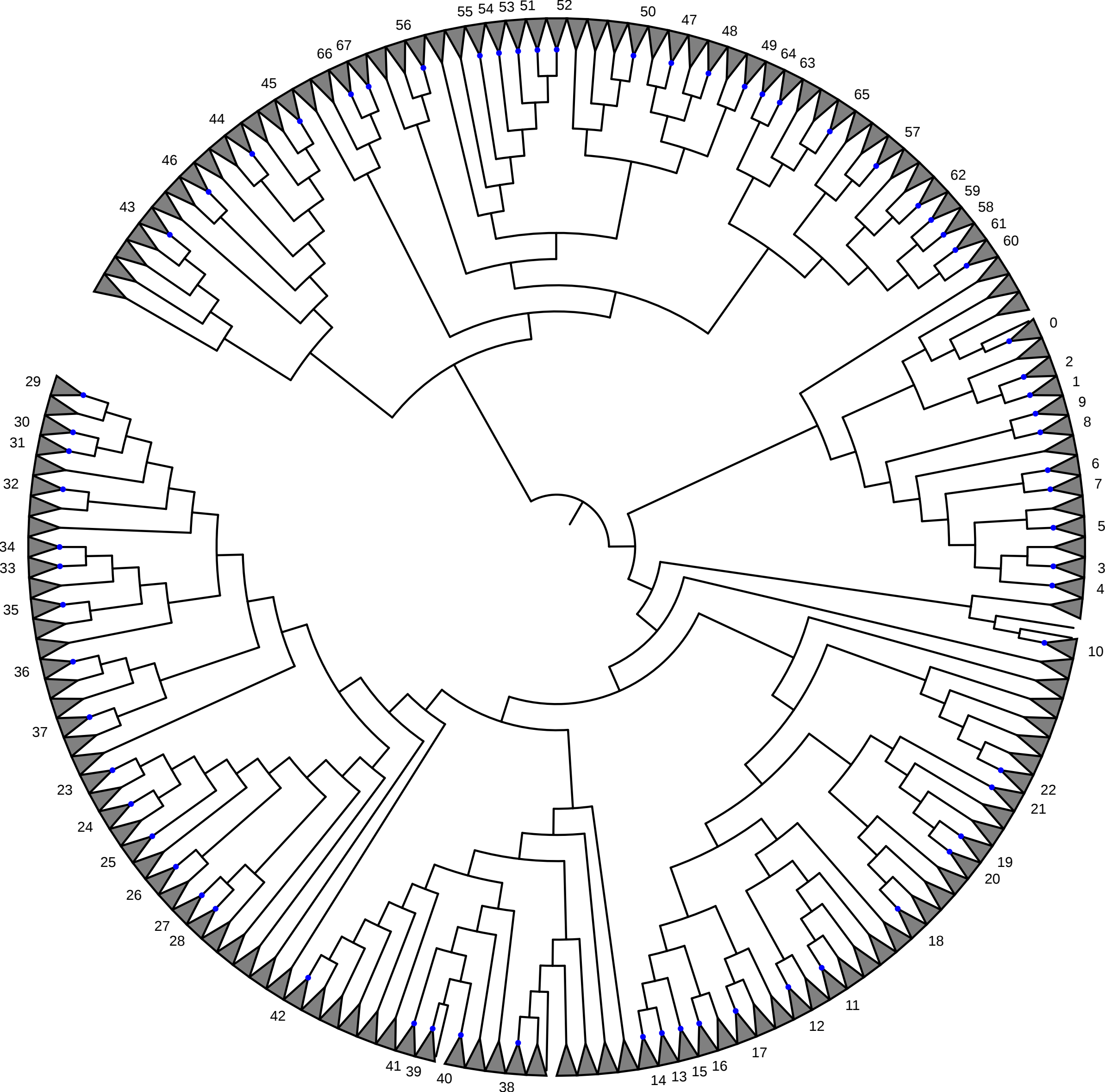

Supplement: Supplementary file 2 — Additional file 2: Figure S1. Phylogenomic reconstruction of 6646 viral genomic sequences. The tree was built through Neighbor-Joining based on Dice distances calculated between viral genomic sequences from both NCBI RefSeq and those reconstructed from metagenomes, fosmid libraries and prophages integrated into prokaryote genomes. The tree was midpoint rooted. To better display higher-order associations between lineages, nodes were collapsed according to their Level-1 lineage assignments or if all the leaves in a node were not assigned to any lineages. [file 12915_2019_723_MOESM2_ESM.pdf]

A

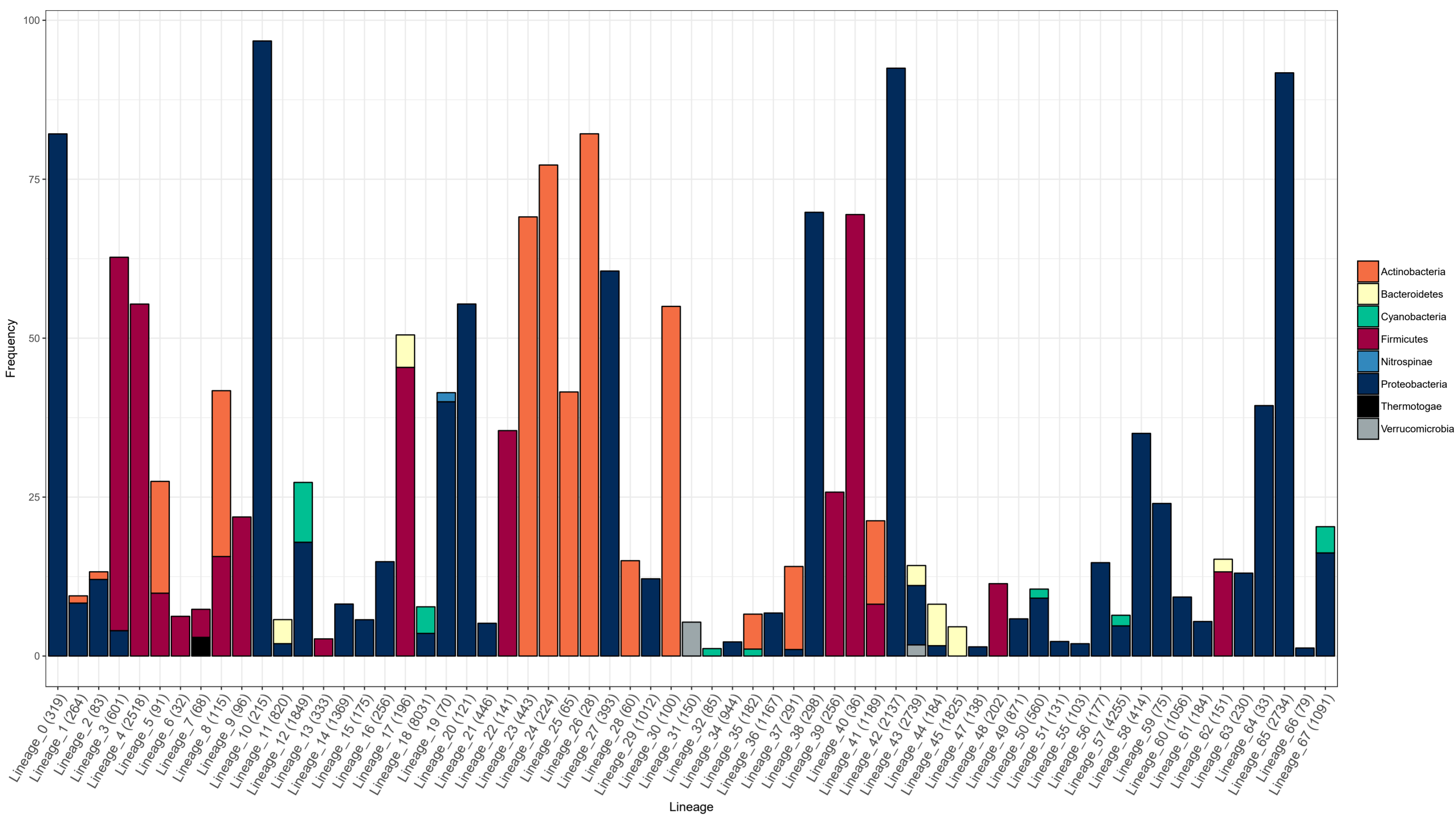

B

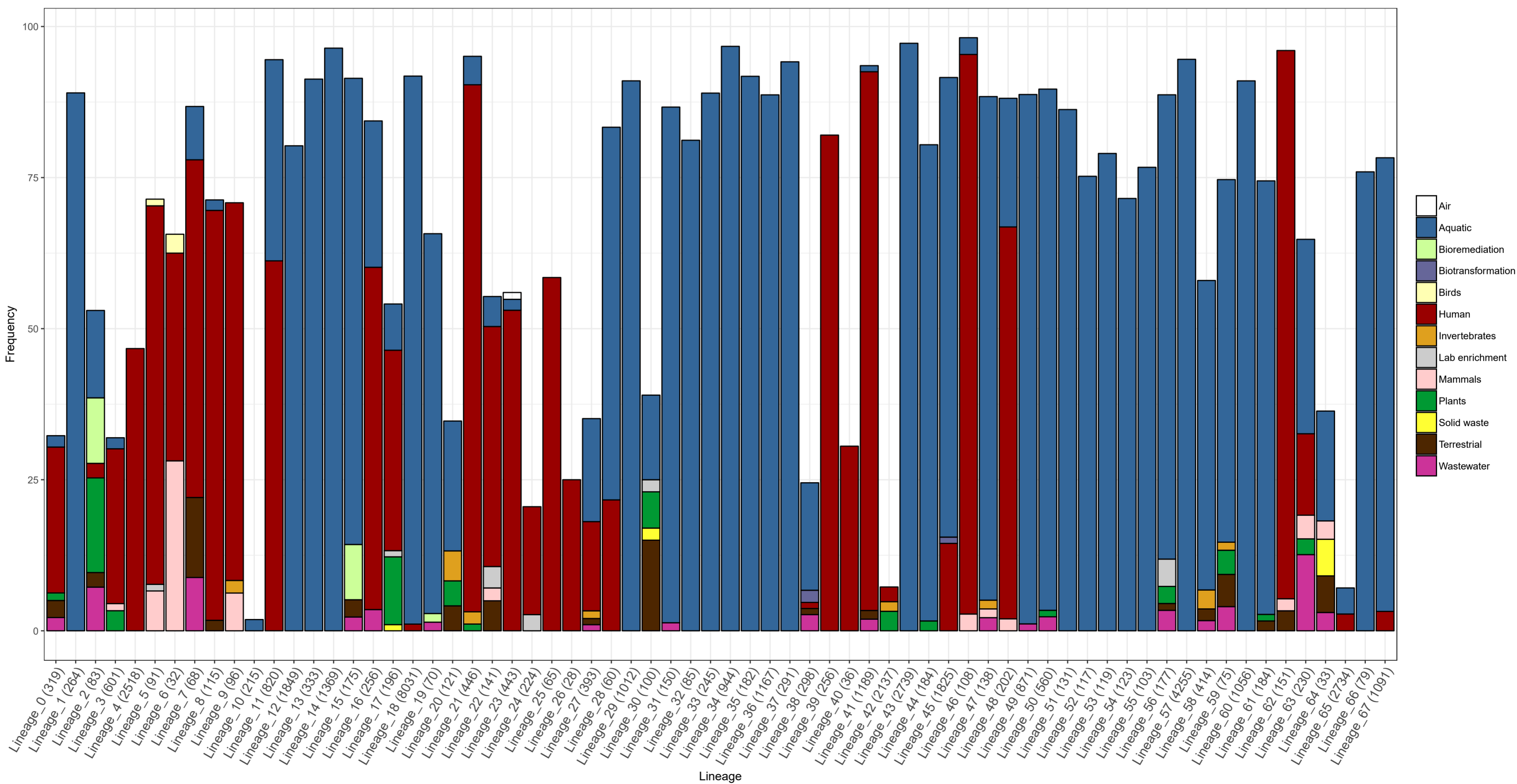

Supplement: Supplementary file 6 — Additional file 6: Figure S4. Prevalence of targeted host and ecosystem sources among Level-1 GL-UVAB lineages assigned through phylogenomic reconstruction and lineage expansion by closest relative identification. A) Frequency of infected host phyla across each of the 68 identified lineages. B) Frequency of ecosystem sources from which viral sequences were obtained across each of the 68 identified lineages. For clarity, only hosts and ecosystems with prevalence within a lineage equal or above 1% are shown. Numbers in parentheses indicate the total number of genomes assigned to each lineage after the step of classification through closest relative identification. [file 12915_2019_723_MOESM6_ESM.pdf]
