# Supplementary material for: Charting the diversity of uncultured viruses of Archaea and Bacteria
Source: BMC Biol. 2019 Dec 29;17:109. doi: 10.1186/s12915-019-0723-8 (PMC6936153; doi:10.1186/s12915-019-0723-8)
Supplement: Supplementary file 3 — Additional file 3: Figure S2. Phylogenomic reconstruction of 2069 genomes of dsDNA viruses of Archaea and Bacteria from RefSeq. The tree was built through Neighbor-Joining based on Dice distances calculated between complete prokaryotic viral genomes from NCBI RefSeq. The tree was midpoint rooted. The innermost ring displays ICTV family level Taxonomic classification, the middle ring displays ICTV subfamily level Taxonomic classification, and the outermost ring displays classifications into lineages identified for the benchmarking dataset. [file 12915_2019_723_MOESM3_ESM.pdf]

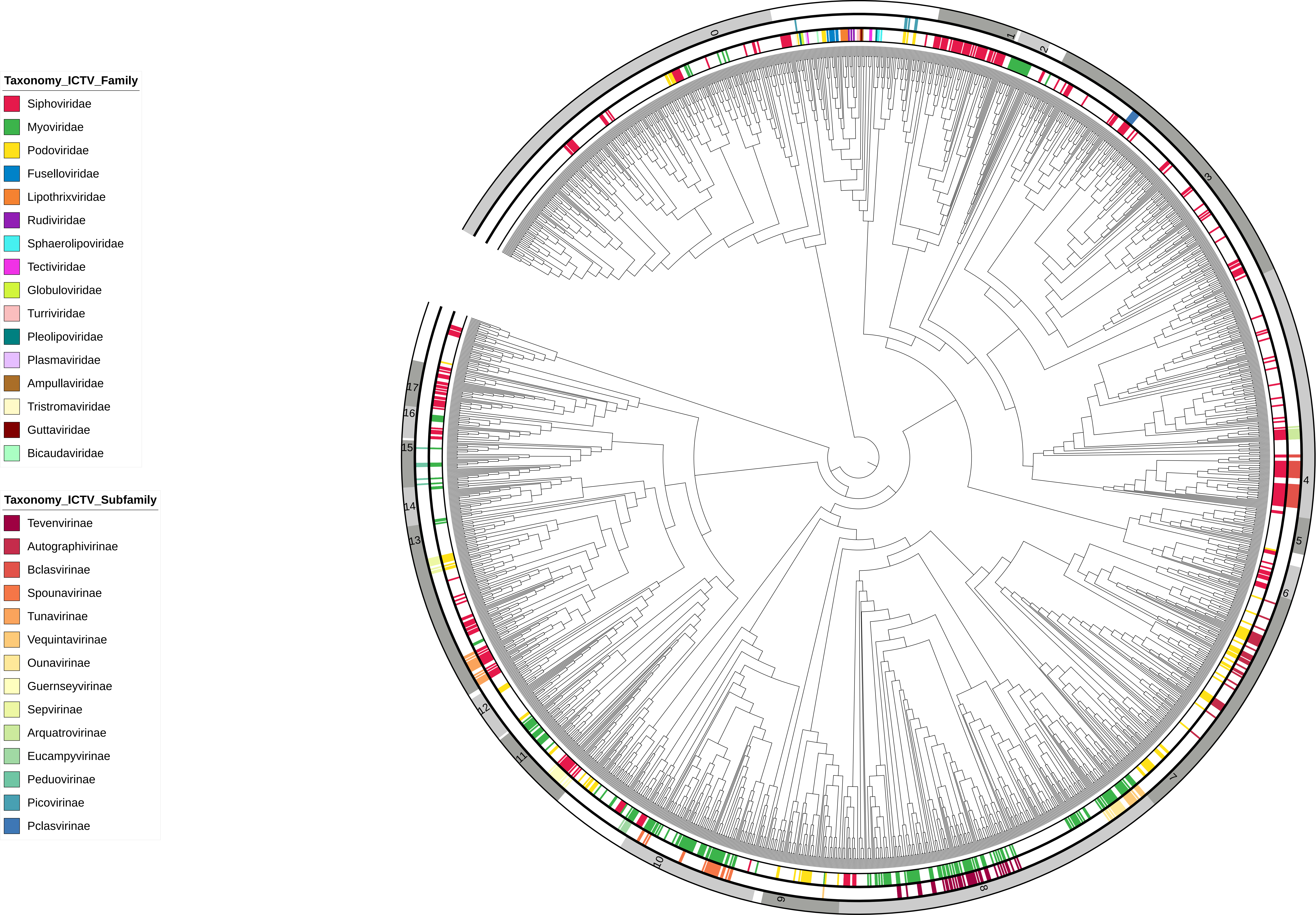
Taxonomy_ICTV_Family
Siphoviridae
Myoviridae
Podoviridae
Fuselloviridae
Lipothrixviridae
Rudiviridae
Sphaerolipoviridae
Tectiviridae
Globuloviridae
Turriviridae
Pleolipoviridae
Plasmaviridae
Ampullaviridae
Tristromaviridae
Guttaviridae
Bicaudaviridae
Taxonomy_ICTV_Subfamily
Tevenvirinae
Autographivirinae
Bclasvirinae
Spounavirinae
Tunavirinae
Vequintavirinae
Ounavirinae
Guernseyvirinae
Sepvirinae
Arquatrovirinae
Eucampyvirinae
Peduovirinae
Picovirinae
Pclasvirinae
0
1
2
3
4
5
6
7
8
9
10
11
12
13
14
15
16
17
