# Supplementary material for: Charting the diversity of uncultured viruses of Archaea and Bacteria
Source: BMC Biol. 2019 Dec 29;17:109. doi: 10.1186/s12915-019-0723-8 (PMC6936153; doi:10.1186/s12915-019-0723-8)
Supplement: Supplementary file 5 — Additional file 5: Figure S3. Concordance between the ICTV taxonomy and the GL-UVAB classification system. A) Bar plots depicting the prevalence of ICTV family level genome classifications among the Level-1 GL-UVAB lineages. B) Bar plots depicting the prevalence of ICTV sub-family level genome classifications among the Level-2 GL-UVAB lineages. C) Heatmap depicting the prevalence of ICTV genera (columns) level classification among Level-3 GL-UVAB lineages (rows). Within squares are depicted the absolute number genomes from a genus assigned to a given lineage, while the color gradient represents the percentage of genomes from a genus assigned to a given lineage. To facilitate visualization rows and columns were clustered based on euclidean distances. [file 12915_2019_723_MOESM5_ESM.pdf]

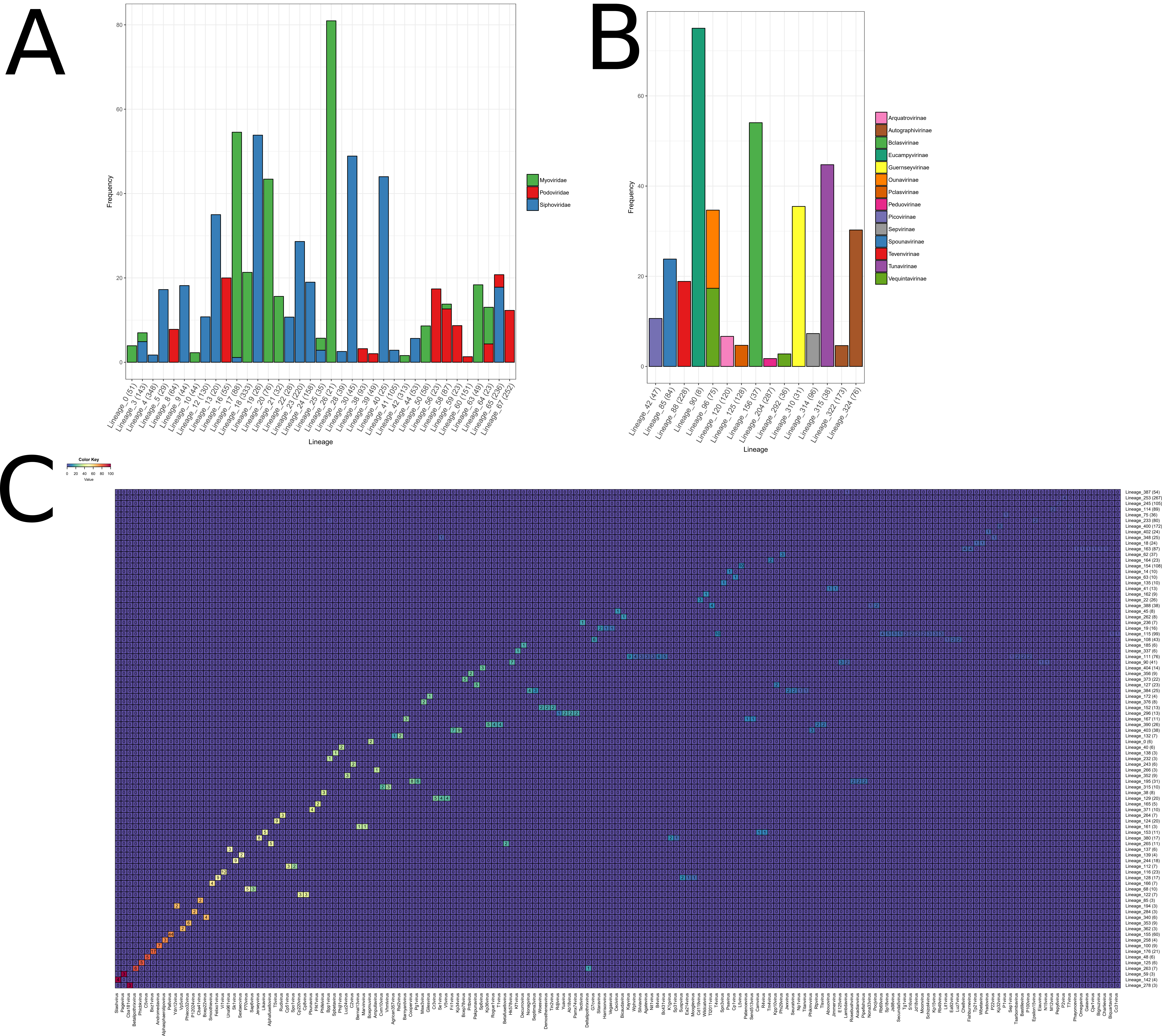
A
Frequency
Lineage
Myoviridae
Podoviridae
Siphoviridae
Lineage_0 (51)
Lineage_3 (143)
Lineage_4 (348)
Lineage_5 (29)
Lineage_8 (64)
Lineage_9 (44)
Lineage_10 (44)
Lineage_12 (130)
Lineage_13 (20)
Lineage_16 (55)
Lineage_17 (88)
Lineage_18 (33)
Lineage_19 (26)
Lineage_20 (76)
Lineage_21 (32)
Lineage_22 (28)
Lineage_23 (220)
Lineage_24 (158)
Lineage_25 (35)
Lineage_26 (21)
Lineage_28 (39)
Lineage_30 (45)
Lineage_38 (93)
Lineage_39 (49)
Lineage_40 (25)
Lineage_41 (105)
Lineage_42 (313)
Lineage_44 (53)
Lineage_50 (58)
Lineage_56 (23)
Lineage_58 (87)
Lineage_59 (23)
Lineage_60 (151)
Lineage_63 (49)
Lineage_64 (23)
Lineage_65 (236)
Lineage_67 (252)
B
Frequency
Lineage
Arquatrovirinae
Autographivirinae
Bclasvirinae
Eucampyvirinae
Guernseyvirinae
Ounavirinae
Pclasvirinae
Peduovirinae
Picovirinae
Sepvirinae
Spounavirinae
Tevenvirinae
Tunavirinae
Vequintavirinae
Lineage_42 (47)
Lineage_85 (84)
Lineage_88 (228)
Lineage_90 (8)
Lineage_96 (75)
Lineage_120 (120)
Lineage_125 (128)
Lineage_156 (37)
Lineage_204 (287)
Lineage_292 (36)
Lineage_310 (31)
Lineage_314 (96)
Lineage_315 (38)
Lineage_322 (173)
Lineage_324 (76)
C
Color Key
Value
